# Prenatal diagnosis of proboscis lateralis

**DOI:** 10.1007/s00404-024-07570-7

**Published:** 2024-06-12

**Authors:** Tania Elger, Cornelia Wiechers, Markus Hoopmann, Karl Oliver Kagan

**Affiliations:** 1 Department of Obstetrics and Gynaecology, University of Tuebingen, Calwerstrasse 7, 72076 Tuebingen, Germany; 2 Department of Neonatology, University of Tuebingen, Tuebingen, Germany

## Text

**Fig. 1:**
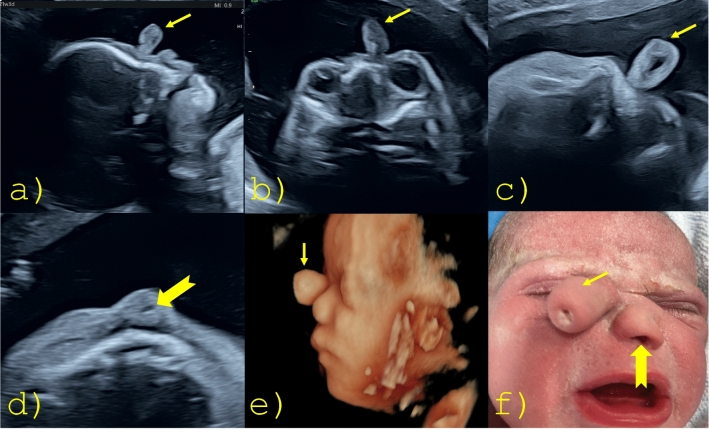
**a** Parasagittal view of the fetal profile, **b** axial view of the orbits, **c** axial view of the PL containing a hypoechoic central portion, **d** transverse view of the single nostril in the nose with heminasal aplasia, **e** 3D image of the side of the fetal face, **f** postnatal photograph of the face. (arrow = proboscis lateralis (PL), notched arrow = a single nostril in the nose with heminasal aplasia)

A 31-year-old woman (gravida 2, para 0) was referred to us at 21 week’s gestation because of an abnormal appearance of the fetal nose on ultrasound. An examination in our department showed a nose with only one nostril and a trunk-shaped mass located next to the nose. The central nervous system and eyes were normal in their ultrasound appearance. There were no other anomalies, especially no further facial abnormalities. The prenatal genetic examinations, including exome analysis, were normal. The fetus was delivered at 38 weeks by elective cesarean section. The female neonate weighed 3330 g. The umbilical artery cord pH was 7.40 and the APGAR score was 9/10/10 at 1, 5, and 10 min, respectively. Postnatal examination showed a blind-ended parasagittal proboscis (proboscis lateralis) measuring approximately 1 cm in length. The neonatal course was uncomplicated, and the baby did not require any respiratory support. The newborn had no other abnormalities (Fig. 1).

Proboscis lateralis (PL) is a soft, finger-like appendage that originates below the forehead and is paramedian in location. [1]. PL was first described by Forster in 1861 in a monograph on congenital malformations of the human body. It is a very rare craniofacial anomaly that is usually unilateral in nature and has an incidence of less than 1:1,000,000 live births [2, 3]. PL may be associated with nasal hypoplasia or heminasal aplasia and holoprosencephaly. It is also associated with other craniofacial abnormalities such as orbital anomalies and cleft lip/palate. However, midline defects such as holoprosencephaly are much less common with PL than in fetuses with a median proboscis. Nonetheless, a detailed ultrasound examination and genetic testing are mandatory in these cases.

Postnatal treatment of PL depends on the associated genetic and/or cerebral findings. In some cases, it includes intensive neonatal care. Amelioration and correction of the defect requires an interdisciplinary approach that includes oral and maxillofacial surgeons to ensure adequate respiration and an acceptable cosmetic outcome [4].

## References

[1] Martin S (2013). Proboscis lateralis. Childs Nerv Syst.

[2] Sakamoto Y (2010). A rare case of proboscis lateralis with median cleft lip. Cleft Palate Craniofac J.

[3] Sarsmaz K (2021). A rare case of bilateral proboscis lateralis: prenatal US and MRI findings. J Clin Ultrasound.

[4] Galiè M (2019). The arrhinias: proboscis lateralis literature review and surgical update. J Craniomaxillofac Surg.

